# Cuproptosis-related gene *LIPT1* as a prognostic indicator in non-small cell lung cancer: Functional involvement and regulation of *ATOX1* expression

**DOI:** 10.17305/bb.2023.9931

**Published:** 2024-06-01

**Authors:** Ruiyun Deng, Lili Zhu, Jun Jiang, Jing Chen, Hua Li

**Affiliations:** 1 Department of Intensive Care Unit, Jiading District Central Hospital Affiliated Shanghai University of Medicine and Health Sciences, Shanghai, China; 2 School of Life Sciences, Fudan University, Shanghai, China; 3 Department of Oncology, Shanghai Jing’an District Central Hospital, Shanghai, China

**Keywords:** Non-small cell lung cancer (NSCLC), cuproptosis-related gene, lipoyltransferase 1 (LIPT1), prognostic indicator, antioxidant 1 (ATOX1)

## Abstract

Non-small cell lung cancer (NSCLC) is a leading cause of cancer-related deaths, necessitating a deeper understanding of novel cell death pathways like cuproptosis. This study explored the relevance of cuproptosis-related genes in NSCLC and their potential prognostic significance. We analyzed the expression of 16 cuproptosis-related genes in 1017 NSCLC tumors and 578 Genotype-Tissue Expression (GTEx) normal samples from The Cancer Genome Atlas (TCGA) to identify significant genes. A risk model and prognostic nomogram were employed to identify the pivotal prognostic gene. Further in vitro experiments were conducted to investigate the functions of the identified genes in NSCLC cell lines. *LIPT1*, a gene encoding the enzyme lipoyltransferase 1, emerged as the central prognostic gene with decreased expression in NSCLC. Importantly, elevated *LIPT1* levels were associated with a favorable prognosis for NSCLC patients. Overexpression of *LIPT1* inhibited cell growth and enhanced apoptosis in NSCLC. We confirmed that *LIPT1* downregulates the copper chaperone gene antioxidant 1 (*ATOX1*), thereby impeding NSCLC progression. Our study identified *LIPT1* as a valuable prognostic biomarker in NSCLC as it elucidates its tumor-inhibitory role through the modulation of ATOX1. These findings offered insights into the potential therapeutic targeting of *LIPT1* in NSCLC, contributing to a deeper understanding of this deadly disease.

## Introduction

About 85% of lung cancer cases are non-small cell lung cancer (NSCLC), highlighting its dominance as the main pathological category [1, 2]. Globally, NSCLC accounts for around 2 million new cases and 1.8 million deaths every year, making it a significant public health concern [3]. The primary etiology of NSCLC, among other factors, includes exposure to cigarette smoke, environmental pollution, and genetic predisposition [4]. Current treatment options for NSCLC consist of surgery, chemotherapy, immunotherapy, and others, whilst the prognosis depends on the stage at diagnosis and the molecular characteristics of the tumor [5, 6]. Despite advances in treatment strategies, the overall five-year survival rate remains relatively low, at approximately 15%–20% [7]. Given the high incidence and mortality of NSCLC, there is an essential need to discover novel targeted therapies concurrently with identifying diagnostic and prognostic markers. Such advancements are essential to mitigate the substantial health burden of NSCLC.

The trace element copper plays an important role in the body’s physiological functions and is involved in the activity of a number of enzymes, especially antioxidant enzymes, which are essential for maintaining normal cellular function and resistance to oxidative stress. Certain cancer types may cause elevated copper levels in the body, as some tumor cells have a greater need for copper, while cancer may prompt the body to release more copper. Patients with NSCLC may face a range of nutrition-related problems, such as loss of appetite, increased consumption, and impaired absorption, which may lead to deficiencies in trace elements such as copper [8]. Cuproptosis, or copper-mediated cell death, was first recognized in 2017 as a distinct mode of cell death, characterized by elevated toxic intracellular copper levels [9]. Unlike other cell death modalities, cuproptosis arises from elevated intracellular copper, which triggers oxidative stress and culminates in cell demise [10]. Recent breakthroughs in oncology emphasize the pivotal role of cuproptosis in both tumor development and therapeutic strategies [11]. For instance, studies on triple-negative breast cancer and glioblastoma cells exhibit increased sensitivity to copper-mediated cell death [12]. This sensitivity can be exploited therapeutically by utilizing copper-chelating agents or modulating copper transporters to selectively induce cuproptosis in cancer cells [13]. In the context of lung cancer, emerging studies suggest that cuproptosis may be important in disease progression and treatment response [14]. Some studies have also demonstrated that lung cancer cells with elevated copper levels are more susceptible to cuproptosis, providing a potential therapeutic target [15]. Moreover, copper chelation therapy has demonstrated a favorable response in preclinical models of lung cancer, further supporting the importance of investigating cuproptosis in this malignancy [16]. Investigating cuproptosis in lung cancer offers potential breakthroughs in therapeutic approaches and deepens insights into the disease’s fundamental biology.

In our study, 16 cuprotosis-related genes were derived from the study by Chi et al. [17]. Through expression analysis, risk modeling, and further assessments, we identified a pivotal prognostic gene linked to NSCLC. We then extensively examined this key gene in NSCLC using survival and clinical feature analyses, immune evaluations, and cellular experiments. Our insights into the molecular mechanisms of these genes in NSCLC illuminated potential therapeutic avenues and prognostic markers, aiming to enhance patient outcomes in NSCLC.

## Materials and methods

### Expression analysis and functional annotation of cuproptosis-related genes in NSCLC

The Cancer Genome Atlas (TCGA) (https://tcga-data.nci.nih.gov/tcga) is a comprehensive resource that provides genomic, epigenomic, transcriptomic, and proteomic data from a wide range of cancer types [18] and GTEx (https://gtexportal.org/) is a valuable database that contains gene expression data from healthy human tissues [19]. First, we analyzed the expression of 16 cuproptosis-related genes (*ATP7A*, *DBT*, *ATP7B*, *DLAT*, *DLST*, *DLD*, *GLS*, *FDX1*, *GCSH*, *LIAS*, *LIPT2*, *LIPT1*, *MTF1*, *NLRP3*, *NFE2L2*, and *PDHB*) in 1017 NSCLC tumor samples in the TCGA and 578 normal samples in the GTEx database. Second, 14 genes that demonstrated significant differential expression (*P* < 0.05) in NSCLC were further examined using the Search Tool for the Retrieval of Interacting Genes (STRING; https://string-db.org/) database and visualized through protein–protein interaction (PPI) networks utilizing the Cytoscape software. For functional enrichment analysis of these genes, the Enrichr database (https://maayanlab.cloud/Enrichr/) was employed to conduct both Gene Ontology (GO) and Kyoto Encyclopedia of Genes and Genomes (KEGG) pathway analysis. The GO annotations included molecular function (MF), cellular component (CC), and biological process (BP). A *P* value below 0.05 was deemed statistically significant.

### Construction of a prognostic risk model for NSCLC using least absolute shrinkage and selection operator (LASSO) regression analysis

For the construction of a prognostic risk model for NSCLC, we preprocessed the gene expression data by normalization and centering. We then performed the LASSO regression method by using the “glmnet” package in R. This regression employed a coordinate gradient descent algorithm to fit the linear model. Optimal tuning parameter determination (*λ*) was achieved through 10-fold cross-validation, aiming for the minimum mean cross-validated error. The chosen optimal *λ* value that minimized the prediction’s mean squared error was set as *λ*_min_ ═ 0.0072. By using this, genes with non-zero coefficients were highlighted, signifying them as potential prognostic biomarkers for NSCLC. The subsequent risk score for each patient was derived using the following formula:

*Risk score* ═ (0.0886 × *ATP*7*B*) + (−0.3104 × *DBT*) + (0.1172 × *DLAT*) + (0.3005 × *DLST*) + (0.2318 × *FDX*1) + (0.009 × *GCSH*) + (−0.0455 × *GLS*) + (0.0149 × *LIAS*) + (−0.1896 × *LIPT*1) + (−0.1781 × *LIPT*2) + (0.0113 × *MTF*1) + (−0.0032 × *NLRP*3) + (0.1026 × *PDHB*).

According to these scores, 1017 NSCLC tumor samples from TCGA were stratified into two groups: high risk (*n* ═ 457) and low risk (*n* ═ 458), based on the average risk score. The Kaplan–Meier survival curves were used to study disease-specific survival (DSS) and the log-rank test was used to determine statistical significance. Differences with a *P* < 0.05 were deemed statistically significant. In addition, a receiver operating characteristic (ROC) analysis was performed to gauge the prognostic accuracy of our model, with the area under the curve (AUC) evaluated at different time points (1 year, 3 years, and 5 years).

### Univariate/multivariate Cox regression analysis for key prognostic gene identification in NSCLC

Univariate Cox regression analysis examines the effect of each predictor independently, while multivariate Cox regression analysis simultaneously considers the impact of multiple predictors on survival [20]. We applied univariate/multivariate Cox proportional hazards regression analysis to evaluate 13 prognostic genes identified in our risk model, along with pertinent clinical variables (age, pT-stage, pN-stage, pM-stage, and smoking history), in TCGA–NSCLC samples. Genes and clinical variables that were shown to be significant in both univariate and multivariate analyses were further investigated. Among them, *LIPT1*, a gene for lipoyltransferase 1 enzyme, was notably represented using a prognostic nomogram for a comprehensive understanding.

### Construction of a prognostic nomogram

We utilized the “rms” package in R software to construct the nomogram. This tool visually represents the predictive model, by assigning numerical values to each prognostic factor based on their contribution to the outcome [21]. These values are summed to derive the total points, which correlate with a specific probability of the clinical outcome. We assessed the nomogram’s accuracy through calibration and validation methods. In the calibrated nomogram, points are near the 45∘ line (calibration plot), which indicates strong agreement between forecasts and observations. Additionally, we performed internal validation using bootstrapping techniques to estimate the concordance index (C-index), which evaluates the nomogram’s discriminative ability.

### Analysis of *LIPT1* expression and its association with clinical features in NSCLC

Initially, we assessed the expression levels of *LIPT1* across 1017 NSCLC tumor samples and 578 normal samples from the TCGA and GTEx databases. By utilizing the Wilcoxon test, we identified a differential expression of *LIPT1* between the tumor and normal samples. The subsequent prognostic evaluation was conducted using the Kaplan–Meier plotter database (http://kmplot.com/analysis/index.php?p=background), examining the influence of *LIPT1* differential expression on overall survival (OS) and progression-free survival (PFS) of NSCLC patients. To further delve into the clinical implications of *LIPT1* expression, we associated its levels with diverse clinical parameters, including gender, age, smoking status, ethnicity, T stage, N stage, M stage, and overall TNM classification. This association was statistically evaluated using the Kruskal–Wallis test, enabling us to discern the significance of *LIPT1* expression across various clinical stratifications. To evaluate statistical significance for differences, *P* < 0.05 were employed.

### Immune analysis in NSCLC based on *LIPT1* expression

Investigating the tumor immune microenvironment (TIME) helps elucidate the mechanisms of tumor progression, immune evasion, and response to immunotherapy [22]. First, we divided 1017 TCGA samples into high-expression (*n* ═ 509) and low-expression (*n* ═ 508) groups according to *LIPT1* expression. Second, we analyzed immune cell infiltration in two groups using the CIBERSORT method (https://cibersort.stanford.edu/) that allowed us to characterize the composition of immune cells in the tumor microenvironment [23]. Furthermore, we employed Spearman correlation analysis to evaluate the link between immune cell infiltration as determined by the EPIC algorithm (http://epic.gfellerlab.org) and *LIPT1* expression in NSCLC. Third, we conducted an ICB response analysis to explore any association between *LIPT1* level and the efficacy of immune checkpoint blockade (ICB) treatment. Finally, we examined the correlation between tumor mutational burden (TMB) and *LIPT1* level by Spearman correlation analysis.

### Cell culture and treatment

We used NSCLC cell lines (NCI-H1299, HCC827, and A549) and normal bronchial epithelial cells (BEAS-2B) from the ATCC. Thermo Fisher Scientific’s humidified incubator (37 ^∘^C, 5% CO_2_) was used for cell cultivation in RPMI-1640 medium (Gibco, Thermo Fisher Scientific) accompanied with a 10% fetal bovine serum (FBS) and 1% penicillin–streptomycin solution. Following cultivation, A549 and HCC827 cells underwent specific treatments. For the investigation of cellular responses to metal ion exposure, cells were treated with 10-µM concentration of copper solutions for 24 h. Simultaneously, a control group of cells was treated with PBS for the same duration to determine any nonspecific cellular reactions.

### Cell transfection

A549 and HCC827 cells were seeded in 6-well plates and cultured for 24 h prior to transfection, allowing them to reach 70%–80% confluency. For overexpression studies, these cells were transfected with either the pcDNA3.1-LIPT1 vector (Eurofins Genomics) or the control empty pcDNA3.1 vector. For knockdown experiments, cells underwent transfection with small interfering RNA targeting antioxidant 1 gene (*ATOX1*) or its respective non-targeting control. All transfections were facilitated using the Lipofectamine 3000 Transfection Reagent (Invitrogen, Thermo Fisher Scientific).

### Quantitative real-time polymerase chain reaction (qRT-PCR)

Total RNA from the samples was extracted using the RNeasy Mini Kit (Qiagen, https://www.qiagen.com/). Subsequent reverse transcription of the extracted total RNA into cDNA was achieved with the iScript cDNA Synthesis Kit (Bio-Rad, https://www.bio-rad.com/). qRT-PCR was performed on a real-time PCR system using the SYBR Green Master Mix (Applied Biosystems, Thermo Fisher Scientific). Specific primers for *LIPT1*, *ATOX1*, and *GAPDH* (which served as an internal control) were utilized. The primer sequences were as follows: *LIPT1* forward, 5’-AGGCTCAGGTCAGAGATGAC-3’, *LIPT1* reverse, 5’-GCTTGATGACGAGGTTGATG-3’; *ATOX1* forward, 5’-CATGCCGAAGCACGAGTT-3’, *ATOX1* reverse 5’-CTTCAGGGTTGCAAGCAGAG-3’; *GAPDH* forward, 5’-TTCAACGGCACAGTCAAGG-3’, *GAPDH* reverse, 5’-CTCAGCACCAGCATCACC-3’.

### Western blotting assay

Cells were lysed using RIPA buffer containing phosphatase and protease inhibitors (Thermo Fisher Scientific). The concentration of protein in the lysates was determined using the BCA Protein Assay Kit (Pierce, Thermo Fisher Scientific). Equal protein amounts were then separated by 10% SDS-PAGE and subsequently transferred to PVDF membranes (Millipore). These membranes were blocked with 5% non-fat milk in TBST for 1 h at room temperature. Overnight incubation at 4 ^∘^C was carried out with primary antibodies against LIPT1, ATOX1, and GAPDH (all from Abcam, 1:2000 dilution). Following washes with TBST, membranes were exposed to HRP-linked secondary antibodies (1:5000, Cell Signaling Technology) for 1 h. Protein bands were visualized using the ECL detection system (Amersham Biosciences) and quantified using ImageJ (NIH), with normalization to GAPDH.

### Cell Counting Kit-8 (CCK-8) assay

For CCK-8 testing, 1×10^4^ cells were seeded into a 96-well plate and incubated for five days, whilst the assessments were performed daily. Each well received 10 µL of CCK-8 reagent from Dojindo Molecular Technologies in Japan at each time point. This was followed by 2-h incubation at 37 ^∘^C. To ascertain cell viability, the absorbance at 450 nm was observed by a microplate reader (BioTek Instruments, USA).

### Transwell assay

In the transwell migration assay, cells suspended in serum-free media were seeded into the upper chamber of a Transwell insert (8-µm pore size, Corning, USA) laced in a 24-well plate. The lower chamber contained media and 10% FBS (Gibco, Thermo Fisher Scientific, USA) as a chemoattractant. The migrated cells under the membrane were fixed with 4% paraformaldehyde (Sigma-Aldrich, USA) for 15 min and stained with 0.1% DAPI (Sigma-Aldrich, USA) for 20 min following incubation. Non-migrated cells in the upper chamber were gently removed using a cotton swab. The stained, migrated cells were then observed and counted under a 200× inverted microscope (Olympus, Japan). The invasion assay was similarly conducted, with the addition of Matrigel in the upper chamber. For both assays, at least five random fields per insert were counted to determine the number of migrated and invading cells.

**Figure 1.**
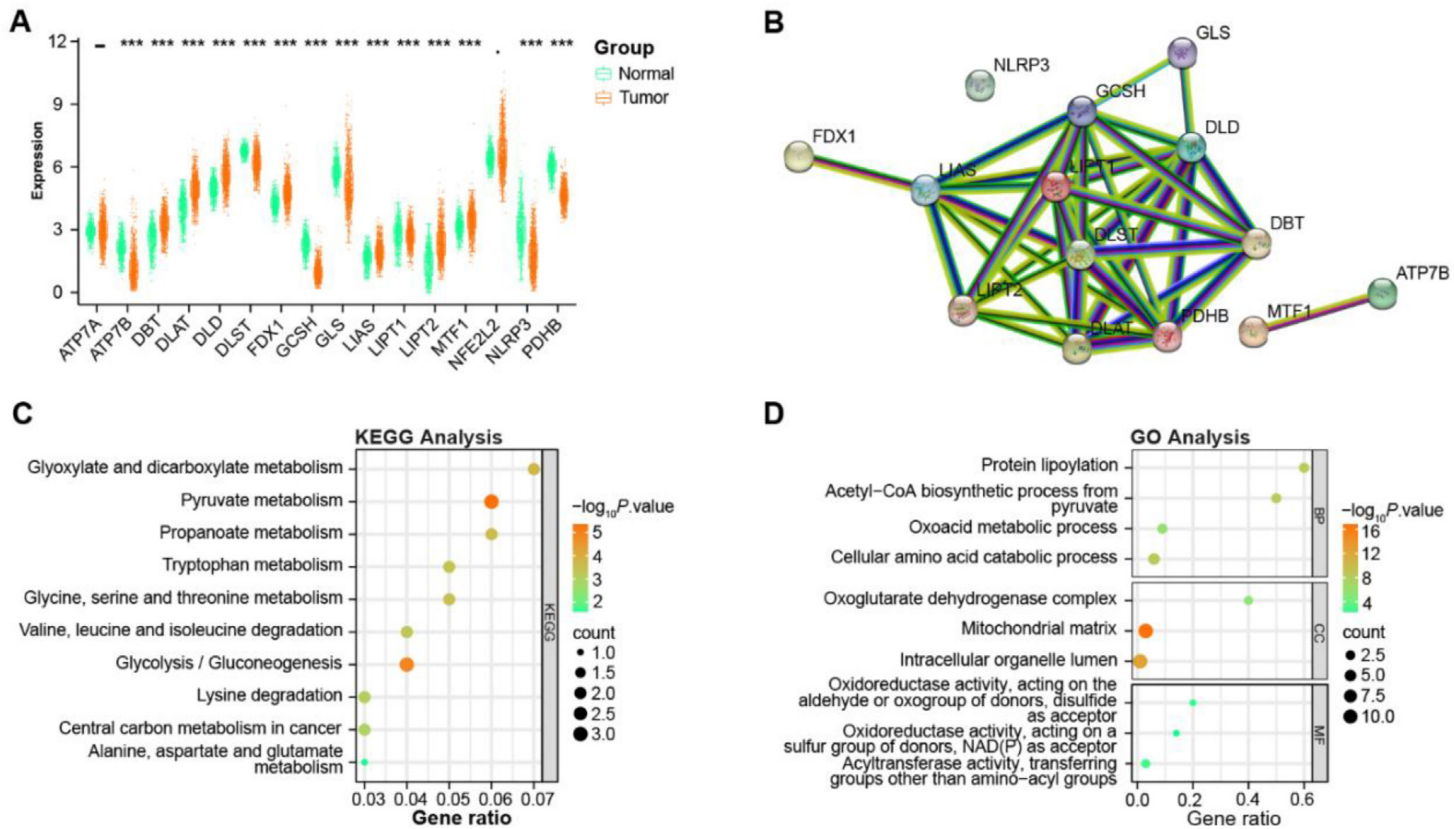
**Differential expression and functional analysis of cuproptosis-related genes in NSCLC**. (A) Expression boxplots of the 16 cuproptosis-related genes in 1017 NSCLC tumor samples from TCGA and 578 normal samples from GTEx; (B) PPI network constructed to explore the connections between 14 significant cuproptosis-related genes with 13 nodes and 38 edges; (C) KEGG pathway analysis on 14 significant cuproptosis-related genes; (D) GO term analysis on 14 significant cuproptosis-related genes, including cellular component (CC), biological process (BP), and molecular function (MF). Bubble size represents the number of genes involved in the respective term and color intensity reflects the adjusted *P*-value. ***P < 0.001. NSCLC: Non-small cell lung cancer; GTEx: Genotype-tissue expression; TCGA: The Cancer Genome Atlas; KEGG: Kyoto Encyclopedia of Genes and Genome; GO: Gene Ontology; PPI: Protein–protein interaction.

### Flow cytometry

Cells were cultured in 6-well plates under the designated experimental conditions. Following treatment, they were collected, resuspended in the kit, and washed twice in cold phosphate-buffered saline (PBS). Next, 100 µL of the cell solution, containing 1×10^5^ cells in a 5-mL culture tube, was mixed with 5 mL of Annexin V-FITC and 5 mL of propidium iodide (PI). After being gently vortexed, the samples were kept at room temperature in the dark for 15 min. Each tube received 400 µL of 1× binding buffer following the incubation, and the samples were then immediately analyzed using a BD FACSCanto™ II Flow Cytometer (BD Biosciences, San Jose, CA, USA). The FlowJo™ program was used for data analysis (BD Biosciences, Ashland, OR, USA).

**Figure 2.**
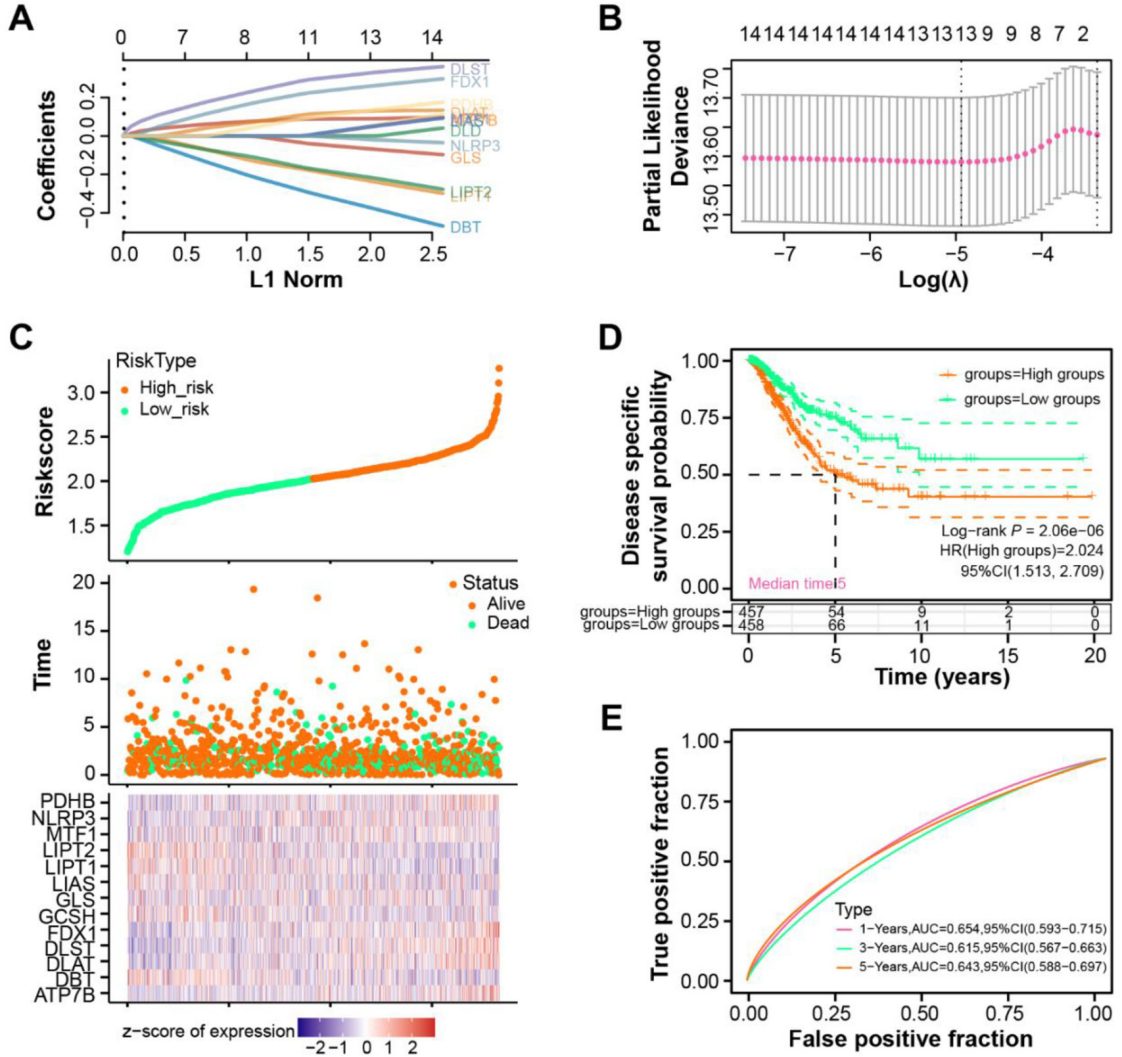
**Thirteen prognostic genes in NSCLC identified in prognostic risk model.** (A) LASSO coefficient profiles of the 14 significant genes. Each curve represents one gene, with the coefficient value plotted against the log(*λ*) sequence, with *λ*_min_ value determined as 0.0072. (B) Tuning parameter (*λ*) selection in the LASSO regression model using 10-fold cross-validation. The optimal *λ* value is determined by the minimum criteria (*λ*_min_), marked by the vertical dashed line. (C) Distribution of the risk scores for the TCGA–NSCLC patients (top). The survival status of patients (middle). The expression heatmap of the 13 prognostic genes (*PDHB*, *NLRP3*, *LIPT2*, *MTF1*, *LIPT1*, *LIAS*, *GLS*, *GCSH*, *FDX1*, *DLST*, *DLAT*, *DBT*, and *ATP7B*) in NSCLC samples (bottom). (D) Kaplan–Meier survival analysis of the high-risk and low-risk groups demonstrates the differences in DSS between the two groups (log-rank test, *P*-value < 0.05). (E) ROC analysis of the prognostic risk model for 1-, 3-, and 5-year OS (0.654, 0.615, and 0.643, respectively). The AUC values represent the predictive accuracy of the risk model for each time point. NSCLC: Non-small cell lung cancer; LASSO: Least absolute shrinkage and selection operator; ROC: Receiver operating characteristic; OS: Overall survival; AUC: Area under the curve; TCGA: The Cancer Genome Atlas; DSS: Disease-specific survival; LIPT1: Lipoyltransferase 1.

### Statistical analysis

Experiments were mostly done in triplicate. Data are shown as mean ± SD. For two-group comparisons, student’s *t*-test was used. For multiple comparisons, we used one-way ANOVA followed by Tukey’s post-hoc test. Survival outcomes were gauged by the Kaplan–Meier method and the log-rank test. Spearman rank determined correlations. A *P* value < 0.05 was deemed significant. SPSS software (version 25.0, IBM, USA) facilitated all analyses.

## Results

### Differential expression and functional analysis of cuproptosis-related genes in NSCLC

In our study, we analyzed the expression patterns of 16 genes associated with cuproptosis in a cohort of 1017 NSCLC tumor samples from TCGA and 578 normal tissue samples from the GTEx database. Among these genes, *ATP7A* and *NFE2L2* did not display statistically significant differences in their expression levels between the tumor and normal samples. Conversely, the analysis revealed that 14 genes exhibited substantial differences in expression between NSCLC and normal tissue samples. These genes are *ATP7B*, *DBT*, *DLD*, *DLAT*, *DLST*, *GCSH*, *FDX1*, *GLS*, *LIPT1*, *LIAS*, *LIPT2*, *NLRP3*, *MTF1*, and *PDHB* (Figure 1A). To further explore these genes, we constructed a PPI network, which highlighted 13 nodes interconnected by 38 edges (Figure 1B). Functionally, these genes correlated with KEGG pathways like pyruvate metabolism and glycolysis/gluconeogenesis (Figure 1C). Furthermore, their associations spanned GO terms, such as mitochondrial matrix, intracellular organelle lumen (CC), and cellular amino acid catabolic process (BP), as well as specific metabolic pathways and processes (MF), as shown in Figure 1D.

### Identifying 13 prognostic genes for NSCLC through risk model

Utilizing LASSO regression on 14 significant genes, we established a *λ*_min_ value of 0.0072 (Figure 2A and 2B). Of these genes, 13 genes were significantly tied to the risk model, with *DLD* being the exception. Expression levels of these 13 prognostic genes in NSCLC samples are illustrated in Figure 2C. Intriguingly, in the DSS analysis, the low-risk group presented a poorer survival outcome compared to the high-risk counterpart (Figure 2D). The ROC results of the risk model yielded AUC values of 0.654, 0.615, and 0.643 for 1-, 3-, and 5-years, respectively (Figure 2E).

### *LIPT1* identified as a key prognostic gene in NSCLC

**Figure 3.**
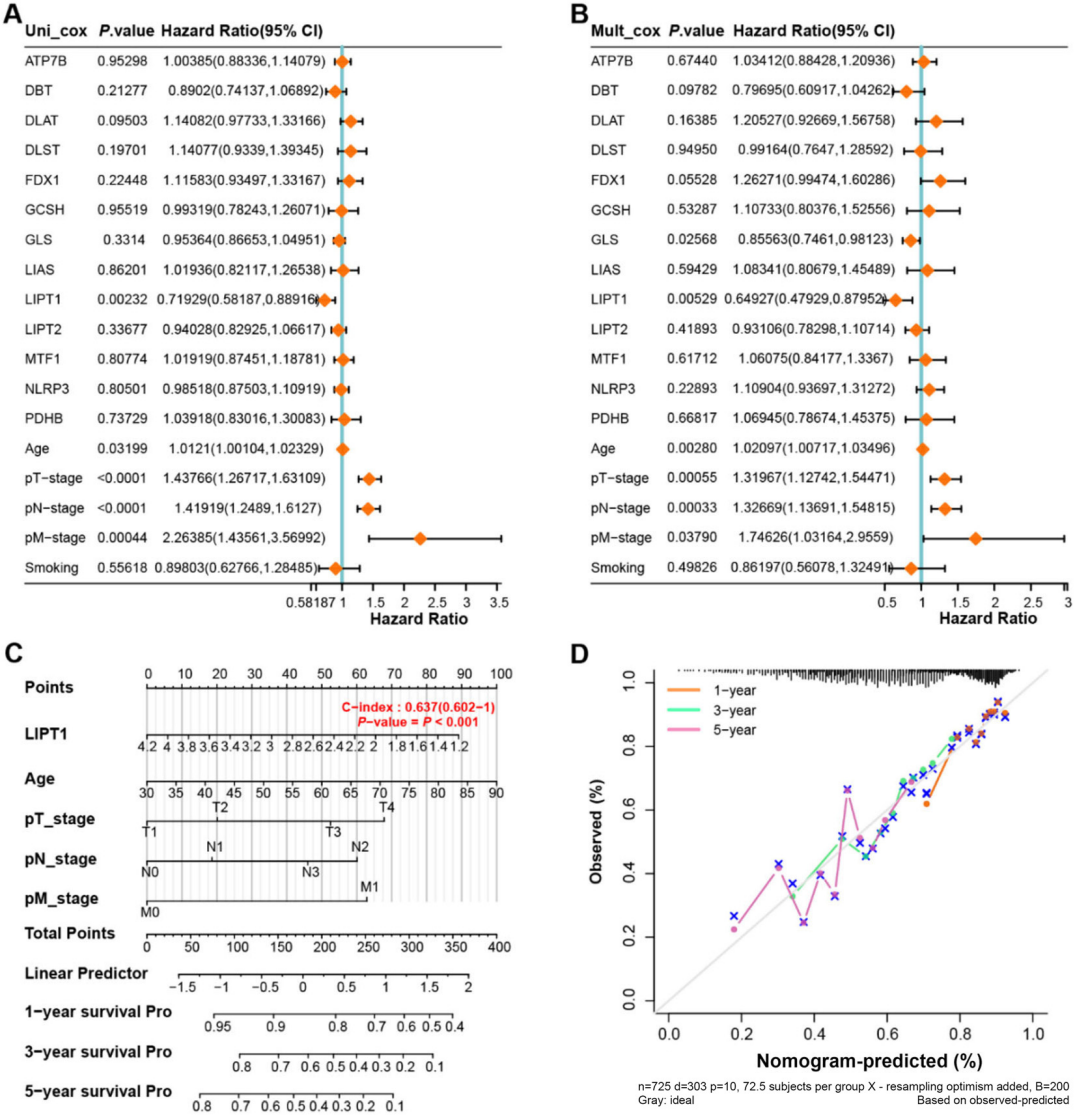
**Identification of key prognostic gene *LIPT1* in the prognostic nomogram of NSCLC.** (A) Univariate Cox analysis of the 13 prognostic genes and clinical features (smoking, age, pT-stage, pN-stage, and pM-stage) of NSCLC patients, with hazard ratios and 95% confidence intervals represented by forest plots; (B) Multivariate Cox analysis of the selected significant genes and clinical features, with hazard ratios and 95% confidence intervals represented by forest plots; (C) Prognostic nomogram integrating *LIPT1* expression and clinical features for predicting 1-, 3-, and 5-year OS rates of NSCLC patients, with each variable assigned a point on the nomogram’s scoring scale; (D) Calibration curves of the nomogram for 1-, 3-, and 5-year OS predictions, indicating the agreement between predicted and actual outcomes, with the *x*-axis representing the predicted survival rates and the *y*-axis representing the observed survival rates. The closer the curve is to the 45-degree line, the better the prediction performance of the nomogram. NSCLC: Non-small cell lung carcinoma; OS: Overall survival; LIPT1: Lipoyltransferase 1.

Combining NSCLC clinical attributes (smoking, age, pT-stage, pN-stage, and pM-stage) with 13 previously mentioned prognostic genes, we executed univariate and multivariate Cox analyses (Figure 3A and 3B). These analyses showed *LIPT1* as the sole gene manifesting significant expression consistently. Thus, *LIPT1* was designated as a primary prognostic gene and incorporated into the NSCLC nomogram (Figure 3C). Calibration results revealed a consistent alignment between anticipated and observed outcomes, especially notable at the 1- and 3-year marks, underscoring *LIPT1*’s substantial prognostic significance in NSCLC (Figure 3D).

### *LIPT1* expression and its prognostic implications in NSCLC

**Figure 4.**
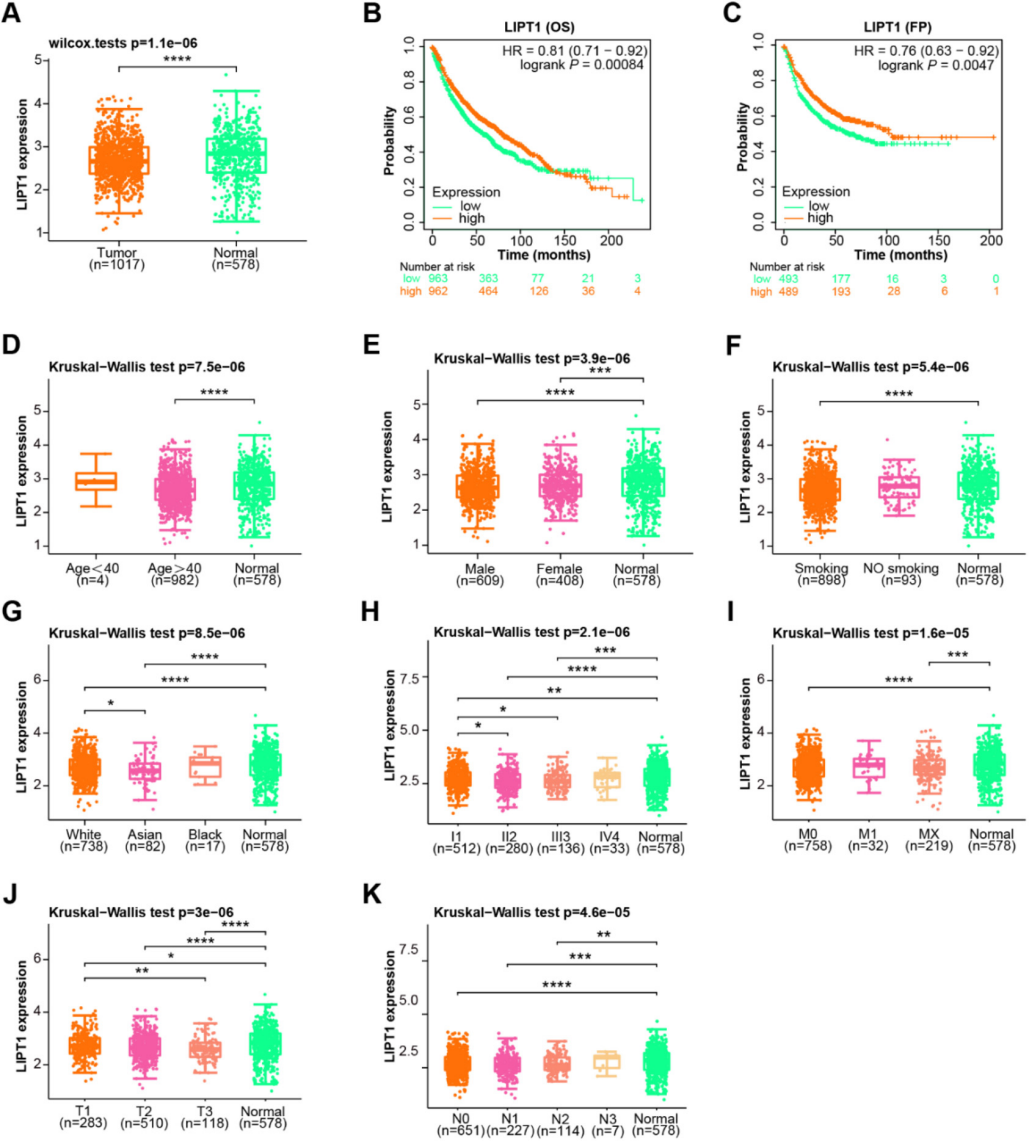
**The association between *LIPT1* expression and prognosis in NSCLC.** (A) *LIPT1* expression levels in 1017 NSCLC tumor samples and 578 GTEx normal samples, with downregulated expression observed in NSCLC; (B) OS analysis of NSCLC patients stratified by high and low *LIPT1* expression levels, with better survival prognosis observed in the high *LIPT1* expression group; (C) PFS analysis of NSCLC patients stratified by high and low *LIPT1* expression levels, with better survival prognosis observed in the high *LIPT1* expression group; (D–K) *LIPT1* expression levels in normal and NSCLC tumor samples based on various clinical characteristics, including age, gender, smoking status, ethnicity, TNM classification, T, N, M stage in order. *P < 0.05, **P < 0.01, ***P < 0.001, ****P < 0.0001. OS: Overall survival, PFS: Progression-free survival; NSCLC: Non-small cell lung cancer; GTEx: Genotype-tissue expression; LIPT1: Lipoyltransferase 1.

Among 1017 NSCLC tumors and 578 GTEx normal samples, *LIPT1* expression was diminished in NSCLC (Figure 4A). The OS and PFS analyses revealed that elevated *LIPT1* levels correlated with improved survival outcomes (Figure 4B and 4C). Detailed subgroup analysis showed that *LIPT1* expression was consistent across different age groups, genders, smoking statuses, and stages (both M and N), showing no significant differences (Figure 4D–4F, 4I, and 4K). However, significant variations were observed in different racial subgroups. Notably, White and Asian populations exhibited marked differences (Figure 4G). Similarly, there were significant disparities in *LIPT1* levels between stages I and II, as well as between stages I and III (Figure 4H). Furthermore, a pronounced difference was evident between T1 and T3 tumor stages (Figure 4J). These observed *LIPT1* expression variations across certain clinical parameters suggest its pivotal role in NSCLC progression and its potential utility as a prognostic marker.

### Association between *LIPT1* expression and altered immune landscape in NSCLC

**Figure 5.**
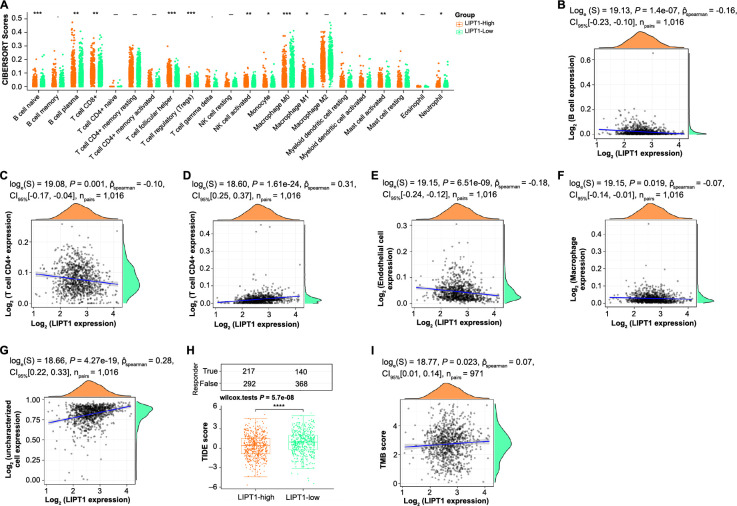
**Immune infiltration and correlation with *LIPT1* expression in NSCLC.** (A) Differential expression of immune cells in TCGA–NSCLC samples with high (*n* ═ 509) and low (*n* ═ 508) *LIPT1* expression using CIBERSORT; (B–G) Spearman correlation analysis between *LIPT1* expression and immune cell infiltration (EPIC) in NSCLC, revealing negative correlation with B cells, CD4+ T cells, endothelial cells, and macrophages, and positive correlation with CD8+ T and uncharacterized cells; (H) TIDE scores in high expression and low expression *LIPT1* groups, with higher scores observed in the low expression group; (I) Spearman correlation analysis between TMB and *LIPT1* expression in NSCLC, showing a positive correlation. *P < 0.05, **P < 0.01, ***P < 0.001, ****P < 0.0001. TCGA: The Cancer Genome Atlas; NSCLC: Non-small cell lung cancer; TMB: Tumor mutational burden; LIPT1: Lipoyltransferase 1.

We stratified TCGA–NSCLC samples into high (509 samples) and low (508 samples) *LIPT1* expression groups. The CIBERSORT analysis highlighted variances in immune cells like naive B cells and plasma B cells (Figure 5A). Spearman correlation for *LIPT1* with the EPIC immune infiltration revealed negative associations with B cells, CD4+ T cells, endothelial cells, and macrophages. Conversely, positive associations emerged with CD8+ T cells and uncharacterized cells (Figure 5B–5G). The high-expression *LIPT1* group manifested a diminished tumor immune dysfunction and escape (TIDE) score (Figure 5H), while a positive correlation was noted between TMB and *LIPT1* expression (Figure 5I). These patterns and correlations suggest an impact of *LIPT1* on immune dynamics in NSCLC.

### *LIPT1* overexpression inhibits migration and invasion of NSCLC cells

**Figure 6.**
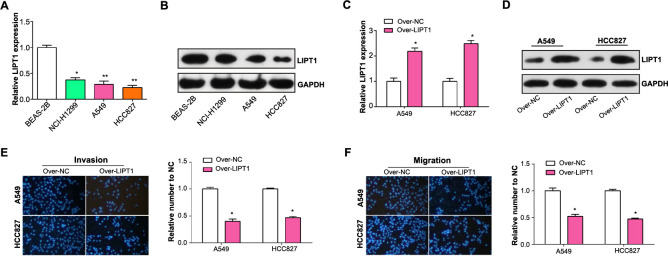
**Experimental validation of *LIPT1* as a tumor suppressor in NSCLC cells.** (A) qRT-PCR analysis showing the relative expression levels of *LIPT1* in normal lung cells and NSCLC cell lines; (B) Western blot analysis confirmed the downregulation of *LIPT1* protein levels in NSCLC cell lines; (C and D) qRT-PCR and western blot analysis confirmed the overexpression of *LIPT1* in NSCLC cell lines; (E) Transwell invasion assay demonstrated a significant decrease in the invasive capacity of HCC827 and A549 cells with LIPT1 overexpression; (F) Transwell migration assay demonstrated a significant decrease in the migratory capacity of HCC827 and A549 cells with LIPT1 overexpression. *P < 0.05, **P < 0.01. NSCLC: Non-small cell lung cancer; qRT-PCR: Quantitative real-time polymerase chain reaction; LIPT1: Lipoyltransferase 1.

qRT-PCR analyses showed that *LIPT1* expression was diminished in NSCLC cell lines, with a notably marked decrease in HCC827 and A549 cell lines when compared to normal lung cells (Figure 6A). The findings from the western blot were also consistent, demonstrating reduced LIPT1 protein levels in these tumor cell lines (Figure 6B). This points to the potential tumor suppressor role of LIPT1 in NSCLC. To delve deeper, we upregulated *LIPT1* in NSCLC cells and validated the overexpression through both qRT-PCR and western blot analysis (Figure 6C and 6D). Interestingly, the increased expression of LIPT1 significantly inhibited the migratory and invasive capabilities of HCC827 and A549 cells. This was evident through transwell assays (Figure 6E and 6F).

### Overexpressed *LIPT1* inhibits copper-stimulated NSCLC cell proliferation and induces apoptosis

**Figure 7.**
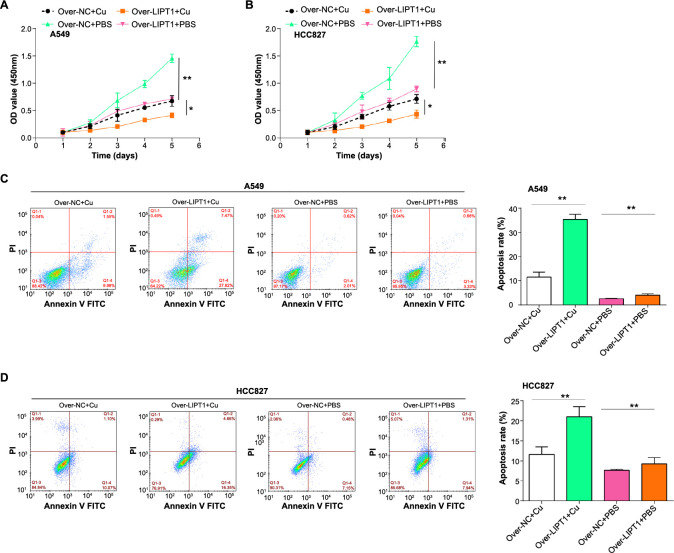
**Effect of copper stimulation on the proliferation and apoptosis in NSCLC cells with *LIPT1* overexpression.** (A and B) CCK-8 assay results showed the effect of *LIPT1* overexpression on the proliferation of HCC827 and A549 cells with or without copper stimulation; (C and D) Flow cytometry results indicated the effect of *LIPT1* overexpression on the apoptosis of HCC827 and A549 cells with or without copper stimulation. *P < 0.05, **P < 0.01. NSCLC: Non-small cell lung cancer; CCK-8: Cell Counting Kit-8; LIPT1: Lipoyltransferase 1.

We investigated the effects of *LIPT1* overexpression on NSCLC cell growth, both with and without copper stimulation, utilizing the CCK-8 assay. Experimentally, we established the following groups: over-NC+Cu, over-LIPT1+Cu, over-NC+PBS, and over-LIPT1+PBS. Notably, the over-LIPT1+PBS and over-LIPT1+Cu conditions suppressed cell proliferation, with the latter exhibiting enhanced inhibition (Figure 7A and 7B). Flow cytometry showed that while over-LIPT1+PBS elevated apoptosis levels, the proapoptotic effect in the over-LIPT1+Cu setup was more pronounced (Figure 7C and 7D). Collectively, our findings underscore that copper stimulation amplifies the inhibitory effects of *LIPT1* overexpression on NSCLC cell growth and its proapoptotic capacities.

### LIPT1 overexpression suppresses NSCLC proliferation via downregulation of *ATOX1* in copper-stimulated conditions

**Figure 8.**
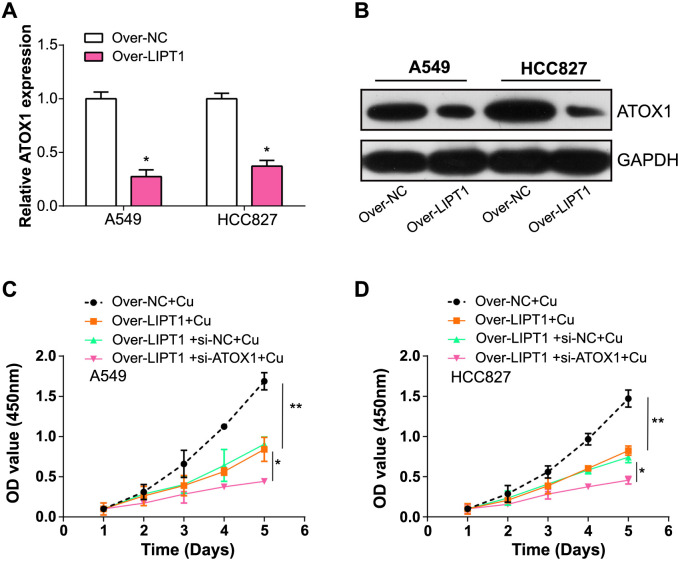
**Cuproptosis-related gene *LIPT1* inhibits the development of NSCLC by inhibiting the expression of copper chaperone gene *ATOX1*.** (A) qRT-PCR analysis of *ATOX1* expression in NSCLC cells with *LIPT1* overexpression; (B) Western blot analysis of ATOX1 protein expression in NSCLC cells with *LIPT1* overexpression; (C and D) CCK-8 assay evaluated the effect of *LIPT1* overexpression and *ATOX1* knockdown on NSCLC cell proliferation after copper stimulation. *P < 0.05, **P < 0.01. NSCLC: Non-small cell lung cancer; ATOX1: Antioxidant 1; qRT-PCR: Quantitative real-time polymerase chain reaction; CCK-8: Cell Counting Kit-8; LIPT1: Lipoyltransferase 1.

Building on earlier findings, we postulated that LIPT1, associated with cuproptosis, impedes NSCLC growth by modulating *ATOX1*, a copper chaperone gene. We presented the results of a PCR assay showing the expression level of *ATOX1* mRNA in NSCLC cells after *LIPT1* overexpression. The data showed a significant negative correlation between LIPT1 overexpression and ATOX1 mRNA levels. This suggests that LIPT1 negatively regulates *ATOX1* transcription, leading to a decrease in *ATOX1* mRNA expression (Figure 8A). This assay analyzed the ATOX1 protein level in NSCLC cells. Consistent with the PCR data, the western blot results indicated that the reduction in ATOX1 protein levels was in response to LIPT1 overexpression. This further supports the idea that LIPT1 plays a regulatory role at the protein level by downregulating *ATOX1* expression (Figure 8B). Additionally, CCK-8 data demonstrated that the combination of copper-induced LIPT1 overexpression and *ATOX1* silencing significantly impeded cell growth (Figure 8C and 8D). The strong inhibition of cell growth under these conditions implies a synergistic effect between copper-induced LIPT1 overexpression and *ATOX1* silencing in curbing NSCLC cell proliferation. These findings reinforced the idea that LIPT1 attenuates NSCLC progression by targeting *ATOX1*.

## Discussion

For NSCLC patients, contemporary diagnostic approaches encompass imaging modalities as well as invasive techniques, such as bronchoscopy, needle biopsy, and mediastinoscopy [24]. The advent of molecular tumor profiling has unveiled targetable genetic aberrations, thereby pioneering a transformative shift in the therapeutic landscape for NSCLC patients [25]. Despite such advances, the 5-year survival rate for advanced-stage NSCLC remains low, at around 15%–20% [26]. In light of these developments, it is imperative to identify potent markers for early NSCLC diagnosis, therapeutic decision making, and prognosis prediction.

In our study, we analyzed the expression of 16 cuproptosis-related genes, as referenced in a prior report, on 1017 NSCLC tumor samples from TCGA and 578 normal samples from GTEx. Of these genes, 14 emerged as significant, showing enrichment predominantly in KEGG pathways, such as pyruvate metabolism, glycolysis/gluconeogenesis, and propanoate metabolism. They also correlated with GO terms, including mitochondrial matrix, cellular amino acid catabolic process, and oxidoreductase activity. Notably, pyruvate metabolism has previously been linked to NSCLC tumorigenesis [27]. Aberrations in pyruvate metabolism within NSCLC cells, particularly characterized by elevated lactate production have been observed [28]. Cancer cells typically exhibit increased glycolysis, a metabolic shift that supports tumor growth, survival, and invasion [29]. Moreover, oxidoreductases, enzymes that catalyze redox reactions, have been implicated in NSCLC pathogenesis [30]. For instance, the upregulation of the oxidoreductase enzyme, thioredoxin reductase 1 (TXNRD1), has been reported in NSCLC, and its inhibition can induce cell cycle arrest and apoptosis [31]. The investigation of these pathways and GO terms in NSCLC could unveil important details about potential molecular mechanisms and identify novel therapeutic targets for this challenging disease.

Subsequently, we used 14 noteworthy genes to construct a prognostic risk model and develop a prognostic nomogram. This nomogram, centered on *LIPT1*, showcased superior predictive accuracy and led us to pinpoint *LIPT1* as the primary prognostic gene in our investigation. LIPT1, or lipoate-protein ligase 1, is an enzyme integral to the lipoic acid metabolic pathway, playing a pivotal role in the efficacy of several vital metabolic enzymes [32]. Previous research on LIPT1 is scarce. Zhang et al. [33] suggested that downregulation of LIPT1 in hepatocellular carcinoma may have a potential tumor-suppressive role. Another study by Li et al. identified LIPT1 as a prognostic biomarker in breast cancer, further supporting its potential role in cancer progression [34].

Our comprehensive assessment, encompassing survival, clinical features, and immune analyses, in conjunction with LIPT1 overexpression experiments, yielded several pivotal insights. Primarily, LIPT1 is underexpressed in NSCLC, and its elevated levels correlate with improved OS and PFS outcomes. Interestingly, factors, such as age, race, and smoking habits, showed no distinct correlation with LIPT1 expression in NSCLC patients. The patterns of immune infiltration hint to LIPT1’s potential role in immune regulation, opening up prospects for innovative immunotherapeutic strategies in NSCLC. Both PCR and western blot assays confirmed the *LIPT1*’s diminished expression and its overexpression was found to robustly inhibit NSCLC cell invasion and migration. Through CCK-8 and apoptosis assays, we observed that copper stimulation augments the inhibitory potency of *LIPT1* overexpression on NSCLC cell proliferation and bolsters its proapoptotic attributes. In essence, *LIPT1* emerges as a prospective tumor suppressor and holds promise as a prognostic marker in NSCLC.

Given the cytotoxicity of excess copper, a complex network of copper chaperones and transport proteins tightly regulates intracellular copper homeostasis [35]. Copper transport within cells is facilitated by tiny molecules called copper chaperones, including cytochrome c oxidase 17, ATOX1, and copper chaperone for superoxide dismutase [36]. In our study, we focused on *ATOX1*, recently identified as relevant to cuproptosis-related in NSCLC [37]. ATOX1 is a small metallochaperone protein that plays a crucial role in intracellular copper trafficking and homeostasis [38]. ATOX1 provides copper to P-type ATPases that transport copper, which are essential for the proper functioning of numerous cellular processes, including redox homeostasis, energy production, and cell signaling [39]. Previous research, including a study by Jana et al. in 2020 suggested that ATOX1 could potentially serve as a prognostic marker and therapeutic target in colorectal cancer [40]. Another study by Blockhuys S et al. in 2021 indicated that ATOX1 might contribute to breast cancer metastasis and could represent a potential therapeutic target [41]. In our experiments using PCR and western blot assays, we examined the effects of *LIPT1* overexpression on ATOX1 levels in NSCLC cells, uncovering an inverse relationship between *LIPT1* overexpression and ATOX1 levels. Further assessments using CCK-8 assays indicated that when *LIPT1* overexpression was stimulated by copper and combined with *ATOX1* knockdown, there was a marked reduction in NSCLC cell proliferation.

## Conclusion

This study pinpointed *LIPT1*, a cuproptosis-related gene, as a pivotal prognostic determinant in NSCLC through bioinformatic methodologies. Demonstrated to function as a tumor suppressor, *LIPT1* presented potential as a diagnostic marker for NSCLC. Notably, *LIPT1*’s putative role in impeding NSCLC progression is hypothesized to be mediated via the modulation of the copper chaperone gene *ATOX1*, a claim substantiated by cellular assays. These insights forged new links between cuproptosis-related genes and NSCLC, enriching avenues for diagnosis, treatment, and prognosis in NSCLC.

## Data Availability

The datasets used and/or analyzed during the current study are available from the corresponding author on reasonable request.
